# Unmasking Celiac Disease Through Chronic Urticaria: Case Report and Scoping Review

**DOI:** 10.3390/nu18030476

**Published:** 2026-02-01

**Authors:** Francesca Cappozzo, Catarina Schrempp Esteves, Fabio Corsolini, Andrea Lacovara, Julieta Pastorino, Matteo Naso, Jacopo Ferro, Federica Malerba, Stefano Bonassi, Marco Crocco

**Affiliations:** 1 Department of Neuroscience, Rehabilitation, Ophthalmology, Genetics, Maternal and Child Health (DINOGMI), University of Genoa, 16146 Genoa, Italy; 5283983@studenti.unige.it (F.C.); 5727889@studenti.unige.it (A.L.); federicamalerba@gaslini.org (F.M.); 2 Centro del Niño y el Adolescente, Hospital CUF Descobertas, 1998-018 Lisboa, Portugal; catarina.schremppesteves@gmail.com; 3 Hematology Unit, IRCCS Istituto Giannina Gaslini, 16146 Genoa, Italy; fabiocorsolini@gaslini.org; 4 Department of Health Sciences, University of Genoa, 16132 Genoa, Italy; julietapastorino@gaslini.org; 5 UOC Direzione Sanitaria, IRCCS Istituto Giannina Gaslini, 16147 Genoa, Italy; 6 UOSD Allergy Center, IRCCS Istituto Giannina Gaslini, 16147 Genoa, Italy; matteonaso@gaslini.org; 7 UOC Pathology Unit, IRCCS Istituto Giannina Gaslini, 16147 Genoa, Italy; jacopoferro@gaslini.org; 8 UOC Pediatria e Neonatologia Imperia, IRCCS Istituto Giannina Gaslini, 18100 Imperia, Italy; 9 Department of Human Sciences and Quality of Life Promotion, San Raffaele University, 00166 Rome, Italy; 10 Clinical and Molecular Epidemiology, IRCCS San Raffaele Roma, 00166 Rome, Italy; 11 Pediatric Gastroenterology and Endoscopy Unit, IRCCS Istituto Giannina Gaslini, 16147 Genoa, Italy

**Keywords:** sprue, urticaria, gluten, autoimmune disease, extraintestinal manifestations, children, dermatitis, food-related disease, dermatologic manifestations

## Abstract

**Background**: Celiac disease (CD) is an immune-mediated, gluten-induced enteropathy with intestinal and extraintestinal manifestations. Chronic urticaria (CU) is a heterogeneous inflammatory skin disorder often considered idiopathic, but emerging evidence suggests possible autoimmune causes. **Methods**: We describe a pediatric case in which CU and angioedema were the sole clinical expressions of CD. We also conducted a scoping review of the literature to assess the prevalence of CD in CU patients and the therapeutic impact of a gluten-free diet (GFD). **Results**: The child’s CU resolved rapidly after initiating a GFD, with complete remission and normalization of anti-tissue transglutaminase at follow-up. Literature review shows that CD is significantly more common in CU patients than in the general population, and several case reports document remission of CU after GFD. However, leading guidelines for CD and CU do not currently recommend mutual screening, and pathophysiological mechanisms linking the two conditions remain incompletely understood. **Conclusions:** Chronic urticaria may be the sole clinical manifestation of CD. Screening for CD in patients with CU may be considered, particularly in those with autoimmune features or disease refractory to standard treatment. Initiating a GFD can lead to rapid symptom remission, reduce dependence on conventional therapies and improve quality of life.

## 1. Introduction

Autoimmune diseases are dramatically increasing worldwide [1]. Celiac disease (CD) is a systemic autoimmune enteropathy triggered by gluten intake in genetically predisposed individuals [2]. Its prevalence has risen significantly over the last 50 years, in part due to improved diagnostic tools and targeted screening of high-risk individuals. Celiac disease prevalence exhibits substantial geographic variability. In the general population, it ranges from 1% to 2%, but higher rates have been reported in certain regions, including Sweden (up to 3%) and the Saharawi population in Algeria (up to 5.6%). Lower prevalence is observed in areas where gluten-containing cereals are not dietary staples and HLA-DQ2/DQ8 alleles are less frequent, such as East Asia and parts of sub-Saharan Africa [3].

Despite its limits, active case-finding—testing individuals with symptoms or associated conditions—is currently the only recommended screening for CD [4]. Updated guidelines from the European Society for Paediatric Gastroenterology, Hepatology and Nutrition (ESPGHAN) and American College of Gastroenterology (ACG) [5,6] recommend screening in patients with conditions at increased risk of autoimmunity, gastrointestinal symptoms or extraintestinal features including specific mucocutaneous manifestations: recurrent aphthous stomatitis and dermatitis herpetiformis (DH). Limited awareness and knowledge of CD among healthcare providers, especially regarding its non-classical presentations, results in underutilization of appropriate serologic screening and delayed referral for further evaluation [7]. A gluten-free diet (GFD) can significantly improve extra-intestinal symptoms in some patients [8].

Dermatitis herpetiformis is the classic gluten-related skin disorder, characterized by granular IgA deposits in the dermal papillae causing pruritic vesicles over extensor surfaces [9]. Atypical manifestations of CD include various dermatologic diseases [10,11,12]. Associations between CD and chronic urticaria (CU), psoriasis, alopecia areata, connective tissue disorders, and atopic dermatitis have been reported [13]. Despite the fact that the prevalence of extraintestinal manifestations of CD in the pediatric population is approximately 60% [14], less is known about the shared pathogenic mechanisms.

Urticaria is characterized by the sudden appearance of itching erythematous wheals, variable in shape and size; in 40% of patients, it is associated with angioedema [15]. The recurrence of wheals, angioedema, or both for longer than 6 weeks defines CU, which has a prevalence of 0.1–1.4% in the general population, and a prevalence in childhood of 0.3–0.8% [16,17]. CU causes severe quality of life impairment and remits in 40–70% of cases within 5 years [18], especially in patients with positive BAT response [19]. The conceptualization of CU as an autoimmune disease is relatively recent, but early observations linked CU with immune dysregulation. In 1962, Rorsman described “antigen-antibody reactions that cause degranulation of leukocytes” in CU [20]. Later studies found associations between CU and thyroid autoantibodies [21]. CU is now classified into type I and type IIb autoimmune phenotypes [22]. Type I endotype (also called autoallergic) is defined by the presence of immunoglobulin E (IgE) autoantibodies directed against self-antigens such as thyroid peroxidase or interleukin (IL) 24. These IgE autoantibodies cross-link the high-affinity IgE receptor (FcεRI) on mast cells, leading to their activation and degranulation, which causes the characteristic wheals and/or angioedema. Patients with this endotype often have normal or elevated total IgE levels and tend to respond well to antihistamines and anti-IgE therapy (e.g., omalizumab). Type IIb endotype is characterized by immunoglobulin G (IgG) autoantibodies targeting either IgE or the FcεRI receptor itself on mast cells and basophils. This leads to direct activation of these cells independent of external allergens. Clinically, type IIb patients often have other autoimmune comorbidities and low total IgE. They frequently show high disease severity with poor response to antihistamines and omalizumab [22].

Despite increasing recognition of extraintestinal manifestations of CD, the available evidence linking CU and CD remains fragmented and heterogeneous. Pediatric-specific data are scarce with discrepancies in the prevalence of CD in children with CU. CU is not currently included among conditions warranting CD screening in leading guidelines [5,6]. Similarly, leading urticaria guidelines do not recommend routine testing for CD, even in patients with CU refractory to standard therapy [15]. This lack of reciprocal consideration reflects a broader absence of consensus on screening strategies, particularly in pediatric populations, where CU is often labeled idiopathic and extensive etiologic investigations are discouraged.

We describe the case of a 4-year-old girl with longstanding CU and angioedema unresponsive to antihistamine therapy, ultimately diagnosed with CD, whose symptoms resolved after GFD. We also review the literature to clarify the association between CD and CU and the potential therapeutic impact of GFD. Due to the heterogeneity of the literature—ranging from case reports to large cohorts, with non-standardized outcomes and diagnostic criteria—a scoping review was chosen because the objectives were exploratory and focused on mapping the available evidence on the association between CU and CD.

## 2. Case Report

**Figure 1:**
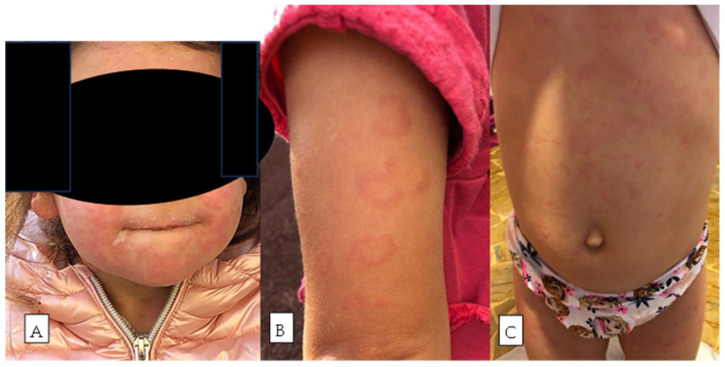
(**A**) Close-up of patient’s face showing widespread red, blotchy patches and areas of dry, peeling skin. (**B**) Red, ring-shaped patches visible on the child’s upper arm. (**C**) Light red, diffuse skin rash spread across the child’s torso.

A 4-year-old girl was referred to our department for evaluation of CU present since age one. She had no gastrointestinal symptoms, growth issues, or family history of urticaria or autoimmune disease. Her urticaria consisted of itchy, migrating wheals over the face, trunk, and limbs, lasting 48–72 h (Figure 1). Episodes increased in frequency, becoming daily and frequently associated with angioedema of the lips and tongue.

Since the onset of symptoms at 1 year of age, the patient had been receiving chronic antihistamine therapy with cetirizine (0.25 mg/kg/day), and oral corticosteroids (betamethasone 0.1 mg/kg) on demand. From 2 years of age, rupatadine (0.2 mg/kg/day) was added due to inadequate symptom control. An allergologic workup showed no triggers based on skin prick testing, ImmunoCAP ISAC test, or Basophil Activation Test (BAT).

Routine evaluation revealed positive CD serology: total IgA 97 mg/dL (normal value 40–200 mg/dL) and anti-transglutaminase (tTG) IgA 40 U/mL (ULN 7–10). Repeat tests confirmed elevated tTG IgA (38 U/mL) and positive endomysial antibodies (EMA) IgA. Hemoglobin, ferritin, and thyroid function were normal, and thyroid autoantibodies were negative. The physical examination during the gastroenterological evaluation was unremarkable. Weight was 15.6 kg (10–25th percentile CDC), height was 96.6 cm (3–5th percentile according to CDC growth charts), growth velocity had been stable in previous years. At diagnosis, inflammatory marker (C-reactive protein (CRP)) was within normal limits. There were no clinical or biochemical signs of infection, and drug-related or physical triggers of urticaria were excluded.

In agreement with the parents, an esophagogastroduodenoscopy (EGD) was scheduled. Six duodenal biopsies showed moderate villous atrophy with crypt hyperplasia and increased intraepithelial lymphocytes (CD3+ ~65/100 enterocytes), Marsh-Oberhuber grade IIIa. Despite a normal macroscopic appearance, histology confirmed CD.

**Figure 2:**
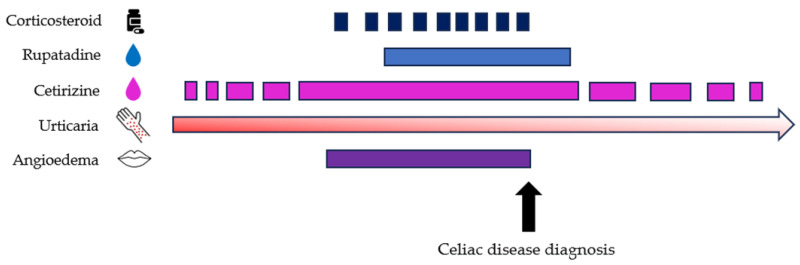
Timeline showing the course of symptoms (urticaria and angioedema) and treatments (corticosteroids, rupatadine, and cetirizine) before and after the diagnosis of celiac disease.

A GFD was initiated with rapid clinical improvement. Only one urticaria episode occurred shortly after starting GFD. Angioedema resolved completely, and corticosteroids were discontinued. Antihistamines were gradually reduced and then withdrawn entirely (Figure 2).

In accordance with current CD guidelines, parents were screened after diagnosis, and both tested negative.

At 6-month follow-up, tTG IgA normalized (3.3 U/mL), parents reported a good adherence to strict GFD. The auxological parameters slightly improved: weight was 16.6 kg (25–50th percentile according to CDC growth charts) and height was 102.8 cm (10–25th percentile according to CDC growth charts). The patient achieved complete clinical remission with substantial improvement in quality of life. At 6 months, a mild elevation of liver enzymes was noted (aspartate aminotransferase 46 U/L, reference < 35 U/L; alanine aminotransferase 39 U/L, reference < 35 U/L), which persisted at 12 months after diagnosis. Protein electrophoresis, cholestasis indices, and inflammatory markers (CRP) were all within normal limits at 6 and 12 months after diagnosis. After two years of GFD, liver enzyme levels normalized.

## 3. Review: Celiac Disease Association with Chronic Urticaria

**Figure 3:**
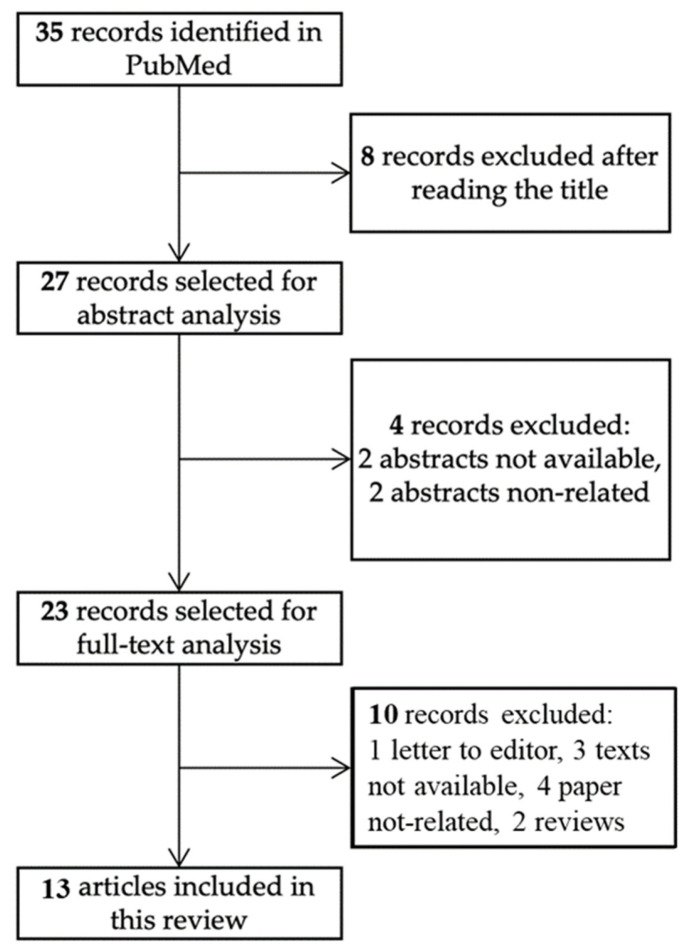
Flow chart of the identified and selected references.

To increase knowledge of the relationship between CD and chronic spontaneous urticaria, authors F.C. (Francesca Cappozzo) and M.C. independently reviewed the literature from the last four decades. The scoping review was conducted with reference to the PRISMA Extension for Scoping Reviews (PRISMA-ScR) guidelines, which were followed wherever possible to enhance transparency and completeness of reporting. After removal of duplicates, two reviewers independently screened titles to assess eligibility. Articles deemed potentially relevant by at least one reviewer were retrieved for abstract analysis and after full-text evaluation, which was performed independently by the same reviewers against predefined inclusion criteria. Any disagreements at either the title/abstract or full-text screening stage were resolved through discussion and consensus; when necessary, a third senior reviewer (S.B.) was consulted. The study selection process is summarized in Figure 3.

The population of interest included children and adults with CU and individuals diagnosed with CD. The review focused on the following concepts: the prevalence of the association between CU and CD; the effect of a GFD on urticaria symptoms. The context included all clinical settings and all study designs, given the heterogeneous and emerging nature of the literature. The PubMed database was searched in March 2024 using the terms “celiac disease” AND “chronic urticaria”. Only English language articles were included. The references of relevant articles were further searched for additional articles. A total of 35 articles were found and 13 articles included, as shown in Figure 3. All variables for which data were searched are listed in Table 1.

### 3.1. Association and Overlap of CD with CU

The association between CD and CU has been progressively explored over the past four decades, evolving from single case reports to large population-based cohort studies.

The first description of CU associated with CD dates to 1987, when Hautekeete et al. reported a middle-aged patient who had CU resolved completely after the diagnosis of CD and initiation of a GFD, suggesting a possible causal relationship between gluten exposure and urticarial symptoms [23]. In 1999, Levine et al. described the first pediatric patient with CD and CU [24]. During the following years, additional case reports supporting the concept of CU as a possible extraintestinal manifestation of CD [25,28,29,30].

The transition from case reports to observational evidence occurred in 2005. Gabrielli et al. conducted a small case–control study in an adult population and did not observe a statistically significant association between idiopathic CU and CD, although the limited number of CD cases substantially restricted the study’s power [26]. In contrast, in the same year, Caminiti et al. investigated a pediatric cohort and reported a significantly higher prevalence of biopsy-confirmed CD (5.0%) among children with CU compared with age-matched controls (0.67%) [27]. These contemporaneous but age-divergent studies underscore the possibility that the association between CU and CD may be more pronounced or clinically relevant in pediatric populations, while also highlighting the methodological constraints imposed by small sample sizes.

More robust evidence emerged from large population-based studies conducted in the 2010s. In 2012, Confino-Cohen et al. analyzed a cohort of 12,778 patients with CU and demonstrated a markedly increased risk of CD (OR 26.96), particularly in female patients. CD was more frequently diagnosed after the onset of CU [31]. One year later, Ludvigsson et al. examined 28,900 individuals with biopsy-proven CD and found a bidirectional association, with an increased risk of developing CU both before and after CD diagnosis (OR ranging from 1.54 to 1.92) [32]. 453 patients with no prior history of urticaria developed the condition, including 79 cases of CU.

More recent studies have provided confirmatory but more conservative estimates. Kosmeri et al. studied 49 children with CU and found CD-specific autoantibodies in 2% of patients, confirmed by duodenal biopsy. Additionally, four children exhibited elevated anti-thyroid peroxidase (anti-TPO) antibody levels, although their thyroid function remained normal [33]. In 2020, consistent with these findings, Lebwohl and colleagues explored the association between CD and urticaria in a large population-based cohort study. After a median follow-up period of 11.4 years, they reported an increased risk of several common skin disorders, including urticaria, among patients with CD compared to the general population (OR, 1.52) [12]. Conversely, a recent Finnish case–control study, including 327 patients with CD, did not find a significant overall association between CD and CU [34].

### 3.2. GFD Effects on CU

The potential therapeutic effect of a GFD on CU in patients with CD has been described progressively over time, primarily through case reports and observational studies.

Several case reports in the literature suggest a beneficial impact of a GFD on CU in patients with CD. The earliest observation in an adult dates back to 1987 [23]. More than a decade later, Levine et al. (1999) described a pediatric patient with CD, CU and thyroid autoimmunity in whom urticaria did not improve despite adherence to a GFD [24].

In the early 2000s, several case reports documented favorable responses to dietary treatment. Candelli et al. (2004) described an adult patient with CD and CU whose urticaria resolved during long-term adherence to a GFD and recurred after gluten reintroduction, supporting a direct role of gluten in symptom maintenance [25]. In 2005, Caminiti et al. reported that all children with concomitant CU and CD included in their case–control study achieved complete remission of urticaria within 5–10 weeks after starting a GFD, while serological normalization occurred more gradually [27]. In the same period, Haussmann et al. (2006) described a young adult with intermittent urticaria resolved completely after CD diagnosis and dietary treatment, even in the absence of specific pharmacological therapy [28].

Subsequent pediatric case reports further strengthened this association. Pedrosa Delgado et al. (2008) reported a young child with cold-induced urticaria and angioedema whose symptoms completely resolved following the GFD [29]. Similarly, Peroni et al. (2010) described a child with subclinical CD presenting with CU as the sole manifestation, with rapid and complete resolution of skin symptoms after dietary treatment [30].

More recent observational data confirms that the response to GFD is not universal. Kosmeri et al. (2019) reported partial improvement of CU symptoms in children with CD-specific autoantibodies after dietary intervention, indicating variable clinical responses [33]. In a Finnish case–control study published in 2023, Turjanmaa et al. observed complete resolution of urticaria in 40% of patients with both CD and CU following GFD [34].

## 4. Discussion

Chronic urticaria is common in patients with CD not in treatment, with a prevalence of 2–5%. Current evidence remains heterogeneous and is predominantly based on observational data. Nonetheless, the literature shows progression from early clinical reports to larger epidemiological studies supporting an association between CU and CD. Several studies indicate that a GFD may lead to partial or complete remission of CU in a subset of patients with CD.

Due to differences in study design and population characteristics, effect sizes vary across studies. However, the consistent observation of increased co-occurrence and clinical improvement after GFD supports a clinical link between the two conditions. In a subset of patients, CU could represent a possible extraintestinal manifestation of CD rather than a coincidental association.

While Italian pediatric guideline for CU recommends screening for CD as part of the diagnostic work-up of CU (Level of evidence V, Strength of recommendation B) [35], current international guidelines for CD and CU do not recommend universal serological mutual screening [5,6,15]. Nevertheless, testing for CD may be considered in selected cases, especially in patients with CU of unknown origin, refractory to standard therapy, or presenting with clinical features suggestive of immune-mediated disease [15].

The accurate identification of cutaneous manifestations, that may represent the only signs of CD, underscores the importance of a collaborative diagnostic strategy among gastroenterologists, allergologists, and dermatologists. This is supported by our case, in which the patient exhibited no gastrointestinal or other manifestations of CD. The sole presenting symptoms were pruritic and migratory wheals, occasionally associated with angioedema.

The strength of evidence supporting an association between CU and CD varies substantially according to study design and age groups. Initial evidence consisted mainly of case reports and small case series, many of which involved pediatric patients, frequently describing resolution of urticaria after initiation of a GFD. Subsequent small case–control studies achieved conflicting results. In particular, two Italian studies reported either no association between CD and CU [26] or a 7.7-fold increased risk of CD among patients with CU [27]. Notably, Caminiti et al. focused on a pediatric population, whereas Gabrielli et al. included only adults. Both studies were limited by the small number of patients with concomitant CD and CU (only five cases), substantially restricting their statistical power.

More robust evidence has emerged from large population-based studies conducted predominantly in adult or mixed-age populations. Confino-Cohen et al. identified 64 patients with both conditions and demonstrated a markedly increased risk of future CD among individuals with CU (OR 27.0) [31]. In contrast, two large Swedish case–control studies reported more modest but still significant associations, with relative risks ranging from 1.5 to 2 [12,32]. These discrepancies in risk magnitude may be partly explained by differences in age distribution and study design. Confino-Cohen et al. relied on serological antibody positivity recorded in an electronic database, whereas the Swedish studies used biopsy-proven villous atrophy from pathology registries. Pediatric studies tend to capture earlier atypical disease presentations, in which CU may represent a prominent or even isolated manifestation of CD, potentially amplifying observed associations. Conversely, adult population-based studies rely largely on registry data, which may underestimate associations with extraintestinal manifestations such as CU. Notably, despite variability in effect size across age groups, all population-based studies consistently reported a higher risk of CD among female patients with CU.

The exact etiopathogenetic relationship between CD and CU remains unclear. In particular, it is uncertain whether CD acts as a triggering factor for CU or if both conditions share a common susceptibility to autoimmune chronic inflammatory processes. CD and DH are autoimmune diseases in which gluten is an environmental trigger that activates a highly specific immune response [36]. Over the past 30 years, the incidence of autoimmune diseases, including CD, DH and CU, have risen sharply globally. This trend suggests that other environmental factors besides gluten may play a role in the development of the disease in genetically susceptible individuals [16,37,38,39]. Notably, children with CD or CU exhibit a higher incidence of various other autoimmune diseases [33,40]. In both diseases, women are more affected than men [41,42]. Kolkhir and colleagues conducted a literature review covering the period from 1992 to 2015 and identified Hashimoto’s thyroiditis as the most prevalent autoimmune disease associated with CU. However, a significant correlation with CD was also observed, with the prevalence of CD in patients with CU ranging from 0.5% to 9.3% [43].

Dermatologist Louis Duhring described DH as a distinct clinical entity four years before Samuel Gee published the first detailed description of CD [44]. Despite the presence of an apparently unremarkable small bowel mucosa in up to 25% of patients [45], patients with DH demonstrate evidence of a celiac-type minor enteropathy. Similar pathogenetic mechanisms may be active in determining the gut damage in DH and CD. In the literature, strong evidence supports an association between CD and DH, recurrent aphthous stomatitis and psoriasis [46]. The associations with other skin conditions (i.e., alopecia, cutaneous vasculitis, vitiligo, lichen, erythema nodosum, pemphigus, atopic dermatitis) are based on case reports and case series, with very few controlled studies [12,26,27,47,48,49,50]. Patients who present only extra-intestinal manifestations, including cutaneous signs, should not be considered as having a mild intestinal disease; they may have a more severe degree of villous atrophy than those presenting with gastrointestinal manifestations [51,52,53], as presented in our case report.

The diagnosis of CU is based on the anamnestic evaluation of the wheals, since there is no diagnostic test to detect this condition: further assessments based on clinical evaluations of the patient should be performed to search for inflammatory diseases, to identify any physical trigger or to exclude genetic or autoimmune diseases [18]. Our patient had excluded the presence of food or inhalant triggers by skin prick testing, and the major allergens were also excluded in the blood by ISAC testing. In addition, the BAT was normal. According to the literature, patients with CU are at increased risk of developing multiorgan autoimmune diseases, including several gastrointestinal autoimmune diseases [31,33]. The hypothesis that an autoimmune pathogenesis may underlie some forms of CU is supported by the observation that intradermal injection of autologous serum elicited immediate wheal-and-flare responses in 60% of patients with spontaneous CU [54].

**Figure 4:**
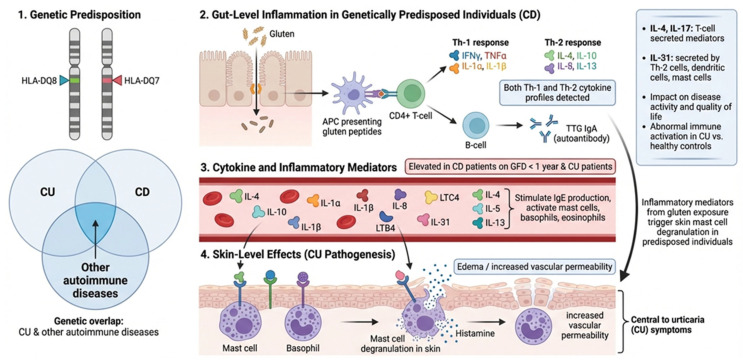
Shared immunopathogenesis of spontaneous chronic urticaria and celiac disease.

Inflammation is a central pathophysiological mechanism in spontaneous CU and CD. A shared genetic predisposition is suggested by common associations with HLA-DQ8 [55] and DQ-7 alleles [56]. Furthermore, a recent genome-wide association study validated the genetic overlap between spontaneous CU and other autoimmune diseases [57]. While CD was historically viewed as an exclusively Th-1-mediated condition, current research indicates a cytokine profile consistent with both Th-1 and Th-2 responses, potentially explaining the observed correlation between the two diseases [58,59]. Patients on a GFD for less than one year exhibited significantly higher levels of both pro-inflammatory and Th-2 cytokines compared to those on a GFD for more than one year [58]. Th-2 cytokines are crucial for stimulating IgE production and activating mast cells, basophils, and eosinophils. A dysregulated activation and degranulation of mast cells and basophils remain central to the increased vascular permeability characteristic of the CU process [59]. A recent study has also revealed abnormal immune system activation and inflammatory responses in CU patients compared to healthy controls, indicating a potential link between the two conditions. Specifically, IL-4 and IL-17 are T-cell-secreted inflammatory mediators, while IL-31 is secreted by Th-2 cells, dendritic cells, and mast cells [60]. The levels of these inflammatory cytokines are directly associated with the pathogenesis, activity, and impact on quality of life in patients with spontaneous CU [61]. This evidence suggest that inflammatory mechanisms triggered by gluten in the gut of genetically predisposed individuals may induce the release of cytokines that promote mast cell degranulation in the skin, thereby triggering CU (Figure 4).

This hypothesis is empirically supported by the observed beneficial effects of a GFD on skin lesions in our reported cases, as well as in other case reports within the literature [23,25,28,29,30]. These findings reinforce the notion that CU may represent a cutaneous manifestation of CD rather than merely a fortuitous association. In both diseases, it has also been hypothesized that viral infection triggers may transiently activate an autoimmune response through processes of molecular mimicry or other immune-mediated mechanisms [19,62,63]. The estimated risk of CD in patients with CU who are not on a GFD is approximately 8–10 times higher than in the general population [27]. Conversely, large-scale studies involving cohorts of children and adults with CD who are on a GFD have shown a slightly elevated frequency of both chronic and acute urticaria compared to healthy controls [31,32]. This further supports a strong correlation between gluten-related inflammation and the risk of CU in this patient population. Given the substantial impact of CU-related pruritus on quality of life [64], identifying its underlying causes is essential to avoid unnecessary treatments and facilitate targeted treatment. The cutaneous manifestations may be the only clinical presentation of CD or may precede the gastrointestinal symptoms, serving as early diagnostic marker of the disease, as demonstrated in our case. In order to prevent diagnostic delays, a GFD should not replace standard CU therapy unless CD is definitively confirmed. Regarding the management of CU in the context of CD, in our patient, the itchy hives did not respond to conventional treatments based on second-generation H1 antihistamines associated with rupatadine. Although a systematic review concludes that there is no basis for using an association of different second-generation H1 antihistamines, this combination is sometimes used in clinical practice as second-line treatment [65]. In addition, the patient received short courses of corticosteroids on demand during severe CU exacerbations with angioedema, in agreement with the guidelines [35], but the only effective benefit was observed after the start of the GFD, resulting in resolving the itchy lesions and preventing angioedema. The clinical remission was very rapid and impressive and the disappearance of urticaria lesion resulted in a significant improvement in quality of life.

This study has some limitations. The database search was restricted to PubMed and a limited number of keywords, and non-English documents were excluded. The available evidence is highly heterogeneous in terms of study design, populations, and diagnostic criteria for both CU and CD, which have evolved substantially over time. Several large studies relied on registry-based diagnoses, raising the possibility of misclassification and limited clinical characterization. In addition, the frequent coexistence of other autoimmune conditions, particularly autoimmune thyroid disease, represents a potential confounding factor. Finally, publication bias—especially among case reports describing favorable responses to a GFD—cannot be excluded. Therefore, the findings should be interpreted with caution.

Nevertheless, based on the data presented by this review, the following conclusions are proposed for their potential clinical implications:

- Screening for coexisting conditions: patients with CU refractory to standard treatment, without an identifiable trigger, should be screened for CD by testing immunoglobulin A level and anti-transglutaminase IgA.
- Monitoring symptoms: in patients with CU the appearance of signs or symptoms consistent with CD should prompt further investigation at each follow-up visit. In cases of clinical uncertainty, serological screening for CD should be repeated.
- Management of CU in CD: after starting a GFD, a complete resolution of CU is possible within a few months. Consequently, it is advisable to consider a gradual reduction in medical treatment for CU as soon as clinically feasible, particularly systemic corticosteroids, to mitigate the risk of growth impairment and optimize catch-up growth in pediatric patients.

## 5. Conclusions

This review highlights a clinically significant association between CU and CD, particularly in children. A substantial body of evidence indicates that CU should be considered as a possible extra-intestinal manifestation of CD, and may often represent the sole presenting symptom, as also illustrated by the presented case. While the exact immune-pathogenetic relationship between CU and CD remains to be fully elucidated, shared genetic predispositions, particularly involving HLA haplotypes, and common inflammatory mechanisms, including cytokine profiles, suggest a deeper connection beyond mere coincidence.

Considering the ‘iceberg’ nature of CD—with many cases remaining undiagnosed—and the significantly increased risk of CD in patients with CU, screening for CD could be considered in selected cases. This is particularly relevant for patients with CU associated with angioedema or autoimmune features, or those refractory to standard treatment without an identifiable trigger, as CU may represent the sole clinical manifestation of CD. Identifying CD in patients with CU of unknown etiology may facilitate targeted treatment, initiating a GFD can lead to a rapid and substantial improvement in CU, potentially allowing for the timely reduction in conventional medical treatments, mitigating associated risks and improving patient quality of life, particularly in children. This underscores the critical need for a collaborative diagnostic approach involving pediatric gastroenterologists, allergologists, and dermatologists to early detect CD in patients presenting with CU.

Overall, the available evidence is limited by bias and heterogeneity. Prospective studies are necessary to establish a definitive correlation between CD and CU and to more comprehensively evaluate the impact of a GFD on CU in pediatric patients with CD. Future research should address existing knowledge gaps including: etiopathogenetic mechanisms linking CD and CU, identifying the inflammatory and immunological pathways involved; investigate potential biomarkers or genetic predispositions that could predict the response of CU to a GFD; explore the role of the gut microbiome and other environmental factors in modulating the immune response that could link CD and CU; examine strategies for improving interdisciplinary collaboration to optimize the early diagnosis and management of patients with co-occurring CU and CD.

## Figures and Tables

**Table 1:** Summary of published case reports and cohort studies evaluating the association between chronic urticaria and celiac disease, including demographic characteristics, study design, odds ratios, treatment approaches, and response of skin symptoms to a gluten-free diet.

Authors	Year	Country	Type of Study	Number of Participants (Sex)	Age in Years, Median (Range) or Mean ± SD	OR *p*-Value (CI)	CU Treatment	Resolution of Skin Symptoms Following a GFD
Hautekeete et al. [23]	1987	Belgium	case report	1 patient (male)	patient 47		H1-antihistamines and H2-antihistamines	complete
Levine et al. [24]	1999	Israel	case report	1 patient (female)	patient 11		H1-antihistamine	no improvement
Candelli et al. [25]	2004	Italy	case report	1 patient (female)	patient 28		H1-antihistamines and corticosteroids	complete
Gabrielli et al. [26]	2005	Italy	case control	80 patients with CU (58 females, 72.5%); 264 controls (192 females, 27.3%)	patients 48 ± 18; controls 45 ± 16	3.3; 0.412 (n.a.)	no data	no data
Caminiti et al. [27]	2005	Italy	case control	79 patients with CU (41 females, 51.9%); 2545 controls	patients 7 (2–18); controls < 19	7.7; 0.0003 (2.3–23.2)	H1-antihistamines, H2-antihistamines and corticosteroids	complete
Haussmann et al. [28]	2006	Canada	case report	1 patient (female)	patient 24		no treatment	complete
Pedrosa Delgado et al. [29]	2008	Spain	case report	1 patient (male)	patient 3		H1-antihistamines	complete
Peroni et al. [30]	2010	Italy	case report	1 patient (female)	patient 9		no treatment	complete
Confino-Cohen et al. [31]	2012	Israel	case control	12,778 patients with CU (8472 female, 66.3%); 10,714 controls (9188 female, 85.7%)	patients 45.3 ± 18.5; controls 44.2 ± 14.2	27.0; <0.0005 (6.6–110.2)	no data	no data
Ludvigsson et al. [32]	2013	Sweden	case control	28,900 patients with CD (17,867 female, 61.8%); 143,397 controls (88,750 female, 61.9%)	patients (30–95); controls (30–95)	1.92; <0.001 (1.5–2.5)	no data	no data
Kosmeri et al. [33]	2019	Greece	cohort	49 patients with CU (23 female, 46.9%)	patients 8.8 (1–15)		no data	partial improvement
Lebwohl et al. [12]	2020	Sweden	case control	43,300 patients with CD (27,022 female, 62.4%); 198,532 controls (123,685 female, 62.3%)	patients 27.1 (0.0–94.9); controls 26 (0.0–95.8)	1.52; <0.05 (1.4–1.6)	no data	no data
Turjanmaa et al. [34]	2023	Finland	case control	327 patients with CD (252 female, 77%); 382 controls (303 female, 79%)	patients 55 (18–86); controls 57 (18–84)	0.5; n.s. (0.2–1.4)	no data	complete in 2/5 (40%) patients with CD and CU

CD: Celiac disease; CU: Chronic urticarial; GFD: Gluten-free diet; CI: Confidence interval; OR: odds ratios; SD: Standard deviation; n.a.: Not available; n.s.: Not significant.

## Data Availability

The original contributions presented in the study are included in the article, further inquiries can be directed to the corresponding authors.

## Abbreviations

CD	Celiac disease
CU	Chronic urticaria
GFD	Gluten-free diet
DH	Dermatitis herpetiformis
ESPGHAN	European Society for Paediatric Gastroenterology, Hepatology and Nutrition
ACG	American College of Gastroenterology
FcεRI	High-affinity IgE receptor
IgE	Immunoglobulin E
IgG	Immunoglobulin G
IgA	Immunoglobulin A
tTG	Tissue transglutaminase
EMA	Endomysial antibodies
ULN	Upper limit of normal
EGD	Esophagogastroduodenoscopy
BAT	Basophil activation test
HLA	Human leukocyte antigen
OR	Odds ratio
CI	Confidence interval
PRISMA-ScR	Preferred Reporting Items for Systematic Reviews and Meta-Analyses extension for Scoping Reviews
Th	T helper
IL	Interleukin

## References

[1] Miller F.W. (2023). The Increasing Prevalence of Autoimmunity and Autoimmune Diseases: An Urgent Call to Action for Improved Understanding, Diagnosis, Treatment, and Prevention. Curr. Opin. Immunol..

[2] Doyle J.B., Silvester J., Ludvigsson J.F., Lebwohl B. (2025). Advances in the Pathophysiology, Diagnosis, and Management of Celiac Disease. BMJ.

[3] Gatti S., Rubio-Tapia A., Makharia G., Catassi C. (2024). Patient and Community Health Global Burden in a World With More Celiac Disease. Gastroenterology.

[4] Catassi G.N., Pjetraj D., Gatti S., Lionetti E., Catassi C. (2023). Celiac Disease Detection Strategies: Poor Performance of the Case-Finding Policy. Am. J. Gastroenterol..

[5] Husby S., Koletzko S., Korponay-Szabó I., Kurppa K., Mearin M.L., Ribes-Koninckx C., Shamir R., Troncone R., Auricchio R., Castillejo G. (2020). European Society Paediatric Gastroenterology, Hepatology and Nutrition Guidelines for Diagnosing Coeliac Disease 2020. J. Pediatr. Gastroenterol. Nutr..

[6] Rubio-Tapia A., Hill I.D., Semrad C., Kelly C.P., Greer K.B., Limketkai B.N., Lebwohl B. (2023). American College of Gastroenterology Guidelines Update: Diagnosis and Management of Celiac Disease. Am. J. Gastroenterol..

[7] Bianchi P.I., Lenti M.V., Petrucci C., Gambini G., Aronico N., Varallo M., Rossi C.M., Pozzi E., Groppali E., Siccardo F. (2024). Diagnostic Delay of Celiac Disease in Childhood. JAMA Netw. Open.

[8] Rodrigo L., Beteta-Gorriti V., Alvarez N., Gómez de Castro C., de Dios A., Palacios L., Santos-Juanes J. (2018). Cutaneous and Mucosal Manifestations Associated with Celiac Disease. Nutrients.

[9] Caproni M., Antiga E., Melani L., Fabbri P., Italian Group for Cutaneous Immunopathology (2009). Guidelines for the Diagnosis and Treatment of Dermatitis Herpetiformis. J. Eur. Acad. Dermatol. Venereol..

[10] Leffler D.A., Green P.H.R., Fasano A. (2015). Extraintestinal Manifestations of Coeliac Disease. Nat. Rev. Gastroenterol. Hepatol..

[11] Ancona S., Bianchin S., Zampatti N., Nosratian V., Bigatti C., Ferro J., Trambaiolo Antonelli C., Viglizzo G., Gandullia P., Malerba F. (2023). Cutaneous Disorders Masking Celiac Disease: Case Report and Mini Review with Proposal for a Practical Clinical Approach. Nutrients.

[12] Lebwohl B., Söderling J., Roelstraete B., Lebwohl M.G., Green P.H.R., Ludvigsson J.F. (2021). Risk of Skin Disorders in Patients with Celiac Disease: A Population-Based Cohort Study. J. Am. Acad. Dermatol..

[13] Therrien A., Kelly C.P., Silvester J.A. (2020). Celiac Disease: Extraintestinal Manifestations and Associated Conditions. J. Clin. Gastroenterol..

[14] Jericho H., Sansotta N., Guandalini S. (2017). Extraintestinal Manifestations of Celiac Disease: Effectiveness of the Gluten-Free Diet. J. Pediatr. Gastroenterol. Nutr..

[15] Zuberbier T., Abdul Latiff A.H., Abuzakouk M., Aquilina S., Asero R., Baker D., Ballmer-Weber B., Bangert C., Ben-Shoshan M., Bernstein J.A. (2022). The International EAACI/GA2LEN/EuroGuiDerm/APAAACI Guideline for the Definition, Classification, Diagnosis, and Management of Urticaria. Allergy.

[16] Fricke J., Ávila G., Keller T., Weller K., Lau S., Maurer M., Zuberbier T., Keil T. (2020). Prevalence of Chronic Urticaria in Children and Adults across the Globe: Systematic Review with Meta-Analysis. Allergy.

[17] Cantarutti A., Donà D., Visentin F., Borgia E., Scamarcia A., Cantarutti L., Peruzzi E., Egan C.G., Villa M., Giaquinto C. (2015). Epidemiology of Frequently Occurring Skin Diseases in Italian Children from 2006 to 2012: A Retrospective, Population-Based Study. Pediatr. Dermatol..

[18] Caffarelli C., Duse M., Martelli A., Calvani M., Cardinale F., Chiappini E., Marseglia G.L., Miraglia Del Giudice M., Tosca M.A., Castagnoli R. (2020). Urticaria in Childhood. Acta Biomed..

[19] Netchiporouk E., Sasseville D., Moreau L., Habel Y., Rahme E., Ben-Shoshan M. (2017). Evaluating Comorbidities, Natural History, and Predictors of Early Resolution in a Cohort of Children With Chronic Urticaria. JAMA Dermatol..

[20] Rorsman H. (1962). Basophilic Leucopenia in Different Forms of Urticaria. Acta Allergol..

[21] Leznoff A., Sussman G.L. (1989). Syndrome of Idiopathic Chronic Urticaria and Angioedema with Thyroid Autoimmunity: A Study of 90 Patients. J. Allergy Clin. Immunol..

[22] Lang D.M., Sheikh J., Joshi S., Bernstein J.A. (2025). Endotypes, Phenotypes, and Biomarkers in Chronic Spontaneous Urticaria: Evolving toward Personalized Medicine. Ann. Allergy Asthma Immunol..

[23] Hautekeete M.L., DeClerck L.S., Stevens W.J. (1987). Chronic Urticaria Associated with Coeliac Disease. Lancet.

[24] Levine A., Dalal I., Bujanover Y. (1999). Celiac Disease Associated with Familial Chronic Urticaria and Thyroid Autoimmunity in a Child. Pediatrics.

[25] Candelli M., Nista E.C., Gabrielli M., Santarelli L., Pignataro G., Cammarota G., Gasbarrini G., Gasbarrini A. (2004). Celiac Disease and Chronic Urticaria Resolution: A Case Report. Dig. Dis. Sci..

[26] Gabrielli M., Candelli M., Cremonini F., Ojetti V., Santarelli L., Nista E.C., Nucera E., Schiavino D., Patriarca G., Gasbarrini G. (2005). Idiopathic Chronic Urticaria and Celiac Disease. Dig. Dis. Sci..

[27] Caminiti L., Passalacqua G., Magazzù G., Comisi F., Vita D., Barberio G., Sferlazzas C., Pajno G.B. (2005). Chronic Urticaria and Associated Coeliac Disease in Children: A Case-Control Study. Pediatr. Allergy Immunol..

[28] Haussmann J., Sekar A. (2006). Chronic Urticaria: A Cutaneous Manifestation of Celiac Disease. Can. J. Gastroenterol..

[29] Pedrosa Delgado M., Martín Muñoz F., Polanco Allué I., Martín Esteban M. (2008). Cold Urticaria and Celiac Disease. J. Investig. Allergol. Clin. Immunol..

[30] Peroni D.G., Paiola G., Tenero L., Fornaro M., Bodini A., Pollini F., Piacentini G.L. (2010). Chronic Urticaria and Celiac Disease: A Case Report. Pediatr. Dermatol..

[31] Confino-Cohen R., Chodick G., Shalev V., Leshno M., Kimhi O., Goldberg A. (2012). Chronic Urticaria and Autoimmunity: Associations Found in a Large Population Study. J. Allergy Clin. Immunol..

[32] Ludvigsson J.F., Lindelöf B., Rashtak S., Rubio-Tapia A., Murray J.A. (2013). Does Urticaria Risk Increase in Patients with Celiac Disease? A Large Population-Based Cohort Study. Eur. J. Dermatol..

[33] Kosmeri C., Siomou E., Challa A., Tsabouri S. (2019). Investigation of Autoimmune Disease in Children with Chronic Spontaneous Urticaria. Int. Arch. Allergy Immunol..

[34] Turjanmaa E., Hervonen K., Huhtala H., Arnala S., Reunala T., Kaukinen K., Salmi T. (2023). Patient-Reported Burden of Skin Disorders in Coeliac Disease. Scand. J. Gastroenterol..

[35] Caffarelli C., Paravati F., El Hachem M., Duse M., Bergamini M., Simeone G., Barbagallo M., Bernardini R., Bottau P., Bugliaro F. (2019). Management of Chronic Urticaria in Children: A Clinical Guideline. Ital. J. Pediatr..

[36] Dunne M.R., Byrne G., Chirdo F.G., Feighery C. (2020). Coeliac Disease Pathogenesis: The Uncertainties of a Well-Known Immune Mediated Disorder. Front. Immunol..

[37] Singh P., Arora A., Strand T.A., Leffler D.A., Catassi C., Green P.H., Kelly C.P., Ahuja V., Makharia G.K. (2018). Global Prevalence of Celiac Disease: Systematic Review and Meta-Analysis. Clin. Gastroenterol. Hepatol..

[38] Okada H., Kuhn C., Feillet H., Bach J.-F. (2010). The “hygiene Hypothesis” for Autoimmune and Allergic Diseases: An Update. Clin. Exp. Immunol..

[39] Stamnaes J., Sollid L.M. (2015). Celiac Disease: Autoimmunity in Response to Food Antigen. Semin. Immunol..

[40] Zingone F., Bai J.C., Cellier C., Ludvigsson J.F. (2024). Celiac Disease-Related Conditions: Who to Test?. Gastroenterology.

[41] Jansson-Knodell C.L., Hujoel I.A., West C.P., Taneja V., Prokop L.J., Rubio-Tapia A., Murray J.A. (2019). Sex Difference in Celiac Disease in Undiagnosed Populations: A Systematic Review and Meta-Analysis. Clin. Gastroenterol. Hepatol..

[42] Liu X., Cao Y., Wang W. (2023). Burden of and Trends in Urticaria Globally, Regionally, and Nationally from 1990 to 2019: Systematic Analysis. JMIR Public Health Surveill..

[43] Kolkhir P., Borzova E., Grattan C., Asero R., Pogorelov D., Maurer M. (2017). Autoimmune Comorbidity in Chronic Spontaneous Urticaria: A Systematic Review. Autoimmun. Rev..

[44] Losowsky M.S. (2008). A History of Coeliac Disease. Dig. Dis..

[45] Mansikka E., Hervonen K., Kaukinen K., Collin P., Huhtala H., Reunala T., Salmi T. (2018). Prognosis of Dermatitis Herpetiformis Patients with and without Villous Atrophy at Diagnosis. Nutrients.

[46] Abenavoli L., Dastoli S., Bennardo L., Boccuto L., Passante M., Silvestri M., Proietti I., Potenza C., Luzza F., Nisticò S.P. (2019). The Skin in Celiac Disease Patients: The Other Side of the Coin. Medicina.

[47] Corazza G.R., Andreani M.L., Venturo N., Bernardi M., Tosti A., Gasbarrini G. (1995). Celiac Disease and Alopecia Areata: Report of a New Association. Gastroenterology.

[48] Naveh Y., Rosenthal E., Ben-Arieh Y., Etzioni A. (1999). Celiac Disease-Associated Alopecia in Childhood. J. Pediatr..

[49] Drago F., Cacciapuoti M., Basso M., Parodi A., Rebora A. (2005). Pemphigus Improving with Gluten-Free Diet. Acta Derm. Venereol..

[50] Ress K., Annus T., Putnik U., Luts K., Uibo R., Uibo O. (2014). Celiac Disease in Children with Atopic Dermatitis. Pediatr. Dermatol..

[51] Nurminen S., Kivelä L., Huhtala H., Kaukinen K., Kurppa K. (2019). Extraintestinal Manifestations Were Common in Children with Coeliac Disease and Were More Prevalent in Patients with More Severe Clinical and Histological Presentation. Acta Paediatr..

[52] Lebwohl B., Kapel R.C., Neugut A.I., Green P.H.R., Genta R.M. (2011). Adherence to Biopsy Guidelines Increases Celiac Disease Diagnosis. Gastrointest. Endosc..

[53] Dickey W., Hughes D. (2001). Disappointing Sensitivity of Endoscopic Markers for Villous Atrophy in a High-Risk Population: Implications for Celiac Disease Diagnosis during Routine Endoscopy. Am. J. Gastroenterol..

[54] Sabroe R.A., Grattan C.E., Francis D.M., Barr R.M., Kobza Black A., Greaves M.W. (1999). The Autologous Serum Skin Test: A Screening Test for Autoantibodies in Chronic Idiopathic Urticaria. Br. J. Dermatol..

[55] O’Donnell B.F., O’Neill C.M., Francis D.M., Niimi N., Barr R.M., Barlow R.J., Kobza Black A., Welsh K.I., Greaves M.W. (1999). Human Leucocyte Antigen Class II Associations in Chronic Idiopathic Urticaria. Br. J. Dermatol..

[56] Bozek A., Krajewska J., Filipowska B., Polanska J., Rachowska R., Grzanka A., Jarzab J. (2010). HLA Status in Patients with Chronic Spontaneous Urticaria. Int. Arch. Allergy Immunol..

[57] Zhang L., Qiu L., Wu J., Qi Y., Gao X., He C., Qi R., Wang H., Yao X., Zhu H. (2023). GWAS of Chronic Spontaneous Urticaria Reveals Genetic Overlap with Autoimmune Diseases, Not Atopic Diseases. J. Investig. Dermatol..

[58] Manavalan J.S., Hernandez L., Shah J.G., Konikkara J., Naiyer A.J., Lee A.R., Ciaccio E., Minaya M.T., Green P.H.R., Bhagat G. (2010). Serum Cytokine Elevations in Celiac Disease: Association with Disease Presentation. Hum. Immunol..

[59] Kolkhir P., Muñoz M., Asero R., Ferrer M., Kocatürk E., Metz M., Xiang Y.-K., Maurer M. (2022). Autoimmune Chronic Spontaneous Urticaria. J. Allergy Clin. Immunol..

[60] Zeng W., Xia J., Zeng Q. (2024). Levels of Serum Inflammatory Cytokines and Their Correlations with Disease Severity in Patients with Chronic Spontaneous Urticaria. Postepy Dermatol. Alergol..

[61] Góra A., Przybył M., Świętochowska E., Machura E. (2022). Assessment of Selected Interleukins (IL-6, IL-17A, IL-18, IL-23) and Chemokines (RANTES, IP-10) in Children with Acute and Chronic Urticaria. Ital. J. Pediatr..

[62] Wedi B., Raap U., Wieczorek D., Kapp A. (2009). Urticaria and Infections. Allergy Asthma Clin. Immunol..

[63] Smatti M.K., Cyprian F.S., Nasrallah G.K., Al Thani A.A., Almishal R.O., Yassine H.M. (2019). Viruses and Autoimmunity: A Review on the Potential Interaction and Molecular Mechanisms. Viruses.

[64] Berberi G., Maazi M., Prosty C., McMullen E.P., Fein M., Lima H., Martinez-Jaramillo E., Ben-Shoshan M., Pourpanah F., Netchiporouk E. (2025). Health-Related Quality of Life in Chronic Urticaria: A Systematic Review and Meta-Analysis. J. Allergy Clin. Immunol. Pract..

[65] Sharma M., Bennett C., Cohen S.N., Carter B. (2014). H1-Antihistamines for Chronic Spontaneous Urticaria. Cochrane Database Syst. Rev..

